# High Incidence of Gastrointestinal Ulceration and Cytogenetic Aberration of Trisomy 8 as Typical Features of Behçet's Disease Associated with Myelodysplastic Syndrome: A Series of 16 Consecutive Chinese Patients from the Shanghai Behçet's Disease Database and Comparison with the Literature

**DOI:** 10.1155/2018/8535091

**Published:** 2018-04-24

**Authors:** Yan Shen, Hai-Fen Ma, Dan Luo, Jian-Fei Cai, Jun Zou, Jian-Long Guan

**Affiliations:** Division of Immunology and Rheumatology, Shanghai Key Laboratory of Clinical Geriatric Medicine, Huadong Hospital, Fudan University, Shanghai 200040, China

## Abstract

This study aimed to investigate the characteristics of Chinese patients with Behçet disease (BD) and myelodysplastic syndrome (MDS) and explore the role played by trisomy 8. This was a retrospective study of patients with BD and MDS from the Shanghai Behçet's disease database who were diagnosed between October 2012 and July 2017. There were 805 patients with BD and 16 also had MDS. Trisomy 8 was examined in patients with BD-MDS and some patients with gastrointestinal (GI) BD. Patients with BD and MDS (16/805; 2%) were more likely to be female and older; display fever and intestinal lesions; have lower leukocyte count, hemoglobin, platelet count; and show higher C-reactive protein and erythrocyte sedimentation rate (ESR) than patients with BD without MDS (all *P* < 0.05). Trisomy 8 was common (81.3%) in patients with BD-MDS. Ulcers in the ileocecal region were more frequently seen in intestinal patients with BD-MDS than in BD without MDS (90.0% versus 48.9%; *P* = 0.032). GI ulceration is common in patients with BD-MDS. Cytogenetic aberrations, especially trisomy 8, may play a role in the pathogenesis of intestinal involvement in patients with BD-MDS.

## 1. Introduction

Behçet disease (BD) is a multisystem inflammatory disorder with mucocutaneous, articular, gastrointestinal, neurologic, and vascular manifestations [1–3]. It typically presents with recurrent oral and genital ulcers and relapsing uveitis [1–3]. BD was first comprehensively described by the dermatologist Hulusi Behçet in 1937. The disease has high prevalence in countries lying along the ancient Silk Road, a route of travel and commerce, extending from the eastern Mediterranean to East Asia [2]. The incidence is higher in East Asia (13.5–20 per 100,000) than in the US and UK (0.12–0.64 per 100,000) [4, 5]. Gastrointestinal (GI) BD is characterized by GI ulcerations. The prevalence of GI BD varies from 3% to 25% in different populations [2, 6–8]. These differences in regional involvement could be due to differences in criteria used to diagnose GI BD and to differences in genetics among various populations [9, 10]. GI involvement can result in life-threatening emergencies such as intestinal perforation and massive bleeding [11].

Myelodysplastic syndrome (MDS) is a blood disorder characterized by impaired generation and maturation of hematopoietic cells in the bone marrow, leading to peripheral blood cytopenia [12]. It may also transform into acute leukemia [12]. Previously, BD and MDS were thought to be two different diseases, but it is now believed that there may be some connection between them since MDS patients can develop autoimmune diseases such as BD or systemic lupus erythematosus, rheumatoid arthritis, relapsing polychondritis, and vasculitis [13–16]. Indeed, most patients with BD or MDS have GI ulcers [17, 18]. In addition, it is unclear whether immunosuppressive agents could play a role in this association [19].

Analysis of a number of case reports has shown an association between trisomy 8 and intestinal BD with MDS [18, 20]. Indeed, trisomy 8 in BD and MDS has been reported in 87% of the patients [18], compared with 7–9% of patients with primary MDS, but without BD [21, 22]. Trisomy 8 with BD but without MDS has also been reported [23, 24].

Many studies have been published about BD and MDS in the Korean and Japanese populations [2, 18, 20, 25–32], but only one such study has been published about Chinese patients [33]. Differences in genetic background, lifestyle, and diet could lead to differences in clinical presentation and outcomes among various populations, even within China. Therefore, the purpose of this retrospective study was to investigate the characteristics of Chinese patients with BD and MDS and explore the role played by trisomy 8.

## 2. Materials and Methods

### 2.1. Study Design

This was a retrospective study of patients with BD and MDS from the Shanghai Behçet's disease database who were diagnosed between October 2012 and July 2017. All patients were with active BD and underwent endoscopic examination. This work was carried out in accordance with the Declaration of Helsinki (2000). The study protocol was approved by the Institutional Review Board of Huadong Hospital. The need for individual consent was waived by the board.

### 2.2. Patients

During the study period, 805 BD patients (with complete clinical and laboratory data) received medical care at the Fudan University Huadong Hospital. All patients were with active BD. At our center, MDS is routinely screened in patients with BD. All patients were diagnosed according to the International Criteria for Behçet Disease (ICBD) [34] (the 1990 criteria were used because the 2014 criteria [35] were stated during the study period). BD was diagnosed in the presence of recurrent oral ulceration and two among the following criteria: (1) recurrent genital ulceration; (2) anterior uveitis, posterior uveitis, cells in vitreous, or retinal vasculitis; and (3) positive pathergy test [34]. MDS was diagnosed and classified according to the WHO classification [36], which takes into consideration morphologic, immunophenotypic, cytogenetic, fluorescence in situ hybridization (FISH) and molecular data.

### 2.3. Literature Search

We searched PubMed with the keyword combination “Behçet's disease OR Behçet's syndrome” AND “myelodysplastic syndrome OR trisomy 8” to identify all cases of BD with MDS reported up to December 2016. Reports with incomplete clinical data were excluded. We collected data related to gender, BD manifestations, age at diagnosis of BD and MDS, GI involvement, and karyotype.

### 2.4. Endoscopy

Endoscopic examination was performed by two experienced gastroenterologists using GIF H260 and CF-H260A1 endoscopes (Olympus, Tokyo, Japan) for upper and lower GI tract examinations, respectively. During endoscopy, two biopsies were taken from the gastric antrum for rapid urease test and histological examination.

### 2.5. Cytogenetics

Bone marrow chromosome analysis was performed using chromosome-banding procedures. At least 20 metaphases were analyzed. Abnormal clones were described according to the 2006 International System for Human Cytogenetic Nomenclature (ISCN) [37], and aberrations were counted following the International Working Group on MDS Cytogenetics (IWGMC) consensus guidelines [38]. FISH analysis was performed on short-term cultured bone marrow. Sample preparations and hybridization were performed according to the manufacturer's recommendations, using commercially available probes (Vysis Inc., Downers Grove, IL, USA). At our hospital, systematic screening for trisomy 8 had been performed on 16 patients with BD and MDS and on 10 patients with GI BD but without MDS. Karyotyping was performed on these patients based on clinical suspicion of trisomy 8. Blood marrow aspiration was done to perform a karyotype, and a minimum of 500 interphase cells were analyzed. If the cells with abnormal signal were <5%, 1000 interphase cells were screened. Normal control values were previously established by using five normal samples of bone marrow donors and 15 bone marrow samples of patients with iron deficiency anemia but with normal karyotype.

### 2.6. Clinical Evaluation and Laboratory Findings

The presence of GI symptoms such as abdominal pain, melena/hematochezia, diarrhea, weight loss, perforation, ileus, or bleeding and extra-GI symptoms such as genital ulcers, uveitis, dermatological lesions, neurological involvement, peripheral vasculitis, and joint involvement at the time of diagnosis was recorded. Erythrocyte sedimentation rate (ESR), C-reactive protein (CRP) level, and peripheral blood counts were also recorded.

### 2.7. Statistical Analysis

Continuous data were tested for normal distribution using the Kolmogorov-Smirnov test. Normally distributed continuous data were presented using mean ± standard deviation and analyzed using Student's *t*-test. Non-normally distributed data were presented as median (range) and analyzed using the Mann–Whitney *U* test. Categorical data were presented as frequencies and analyzed using the chi-square test or Fisher's exact test, as appropriate. Factors found to be significantly different between the groups were entered into a logistic regression model to identify the independently associated factors for the development of MDS. SPSS 20.0 (IBM Corp., Armonk, NY, USA) was used for statistical analyses. Two-sided *P* values < 0.05 were considered statistically significant.

## 3. Results

### 3.1. Characteristics of the Patients

Of the 805 BD patients, there were 16 BD patients with MDS and 789 BD patients without MDS. One female with BD presented with MDS and cervical cancer. Among the 16 patients, four were male and 12 were female. The mean age at diagnosis was 49.9 ± 12.4 years (range: 34–68 years) for BD and 47.5 ± 12.2 years (range: 34–70 years) for MDS. Among the 16 BD patients with MDS, BD was diagnosed prior to MDS in seven (43.8%) patients, after MDS in three (18.7%) patients, and concomitantly with MDS in six (37.5%) patients (Supplementary Table 1).

All patients were clinically and cytogenetically evaluated once when BD was active, with or without treatment. According to the WHO classification, seven patients had MDS-unclassifiable (MDS-U), four had refractory cytopenia with multilineage dysplasia (RCMD), three had refractory anemia with excess of blasts (RAEB), and two had refractory cytopenia with unilineage dysplasia (RCUD). Among the 16 patients with BD and MDS, 13 had abnormal karyotype (81.3%), and all these patients had trisomy 8. Among the 10 patients with BD but without MDS, none were detected with trisomy 8.

### 3.2. Clinical Features of BD with MDS and BD without MDS

Table 1 shows the demographic and clinical features of patients with BD according to MDS. In the BD with MDS group, oral ulcers were the most common finding (100%), followed by genital ulcers (87.5%), GI involvement (62.5%), fever (56.3%), and skin lesions (56.3%). Uveitis and vascular lesions, which are common features of BD, were not recorded in the BD with MDS group. In the univariate analyses, BD with MDS patients were significantly more likely to be female and had older age, fever, and GI involvement than BD without MDS patients (*P* < 0.01) (Table 1). Leukocyte count, hemoglobin levels, and platelet count were significantly lower, and ESR and CRP were significantly higher in BD with MDS patients compared with those with BD without MDS (*P* < 0.05). Logistic regression analysis showed that age (OR = 0.907, 95% CI: 0.829–0.993, *P* = 0.035), GI involvement (OR = 14.349, 95% CI: 1.901–108.319, *P* = 0.010), fever (OR = 1.155, 95% CI: 1.155–339.221, *P* = 0.039), low leukocyte count (OR = 2.841, 95% CI: 1.548–5.214, *P* = 0.001), and high ESR (OR = 0.964, 95% CI: 0.944–0.983, *P* < 0.001) were independently associated with the development of MDS in BD patients.

### 3.3. Characteristics of BD Patients with MDS and GI Involvement

**Figure 1:**
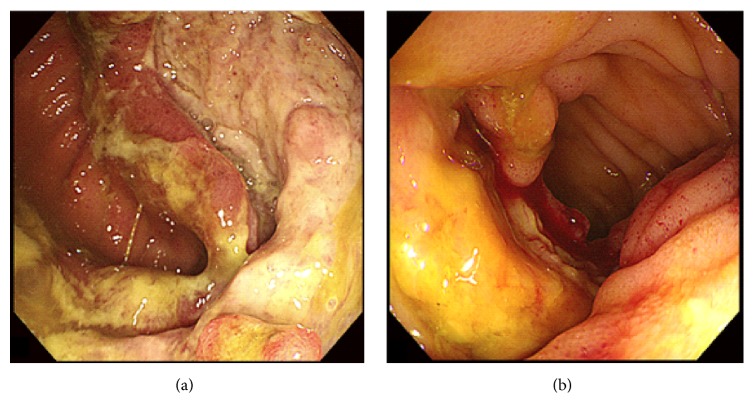
Typical colonoscopic findings of GI involvement in two BD-MDS patients show a severe open ulcer in the ileocecal region (a) and a large, round, deep, and discrete ulcer with edema in the terminal ileum (b).

Among the 805 patients with BD, 94 had GI BD without MDS and 10 had GI BD with MDS.  Table 2 shows the demographic and clinical features of the two groups. In patients with GI BD and MDS, oral ulcers were the most common finding (100%), followed by genital ulcers (80.0%), fever (60.0%), skin lesions (40.0%), positive pathergy test (30.0%), and arthralgia (30.0%). Patients with GI BD and MDS were significantly more likely to have intestinal ulcers in the ileocecal region than GI BD patients without MDS (90.0% versus 48.9%; *P* = 0.032).  Figure 1 shows typical colonoscopy results of patients with GI BD and MDS. In the univariate analyses, patients with GI BD and MDS were more likely to be female and older than patients with BD and MDS without GI (*P* < 0.01) (Table 2). In addition, the former were likely to have lower leukocyte count, hemoglobin level, and platelet count and higher ESR and CRP levels than the latter (*P* < 0.05). There was no statistically significant difference in GI symptoms between the two groups.

### 3.4. BD-Associated MDS with and without Trisomy 8 in Patients with GI BD

There were 10 patients with GI BD and trisomy 8 in our population of 805 patients. For comparison, 10 patients with GI BD but without trisomy 8 were randomly selected among those who underwent bone marrow aspiration for karyotyping.  Table 3 shows the demographic and clinical features of GI BD cases according to trisomy 8. In the univariate analyses, patients with trisomy 8 were more likely to be older than those without trisomy 8 (*P* < 0.01). In addition, the former were more likely to have lower leukocyte count, hemoglobin level, and platelet count and higher ESR than patients without trisomy 8 (*P* < 0.05).

### 3.5. Comparison of Our BD Patients with MDS and Those in the Literature

The literature search yielded 73 articles related to BD with MDS. After excluding reports with incomplete information, 57 patients were available for comparison with our sample [3, 27, 29, 39]. Among the 57 patients, 30 (52.6%) were men. The mean age at diagnosis was 48.7 ± 18.0 years (range: 4–80 years) for BD and 47.4 ± 16.9 years (range: 4–76 years) for MDS. Forty-four (77.2%) patients had trisomy 8, which is comparable with our data. Supplementary Tables 2 and 3 show the demographic and clinical features of our patients and those reported in the literature. In the univariate analyses, there were no significant differences between our patients and those reported in the literature with respect to age at diagnosis of BD, age at diagnosis of MDS, sex ratio, GI involvement, clinical features of BD, and prevalence of trisomy 8.

## 4. Discussion

Many Korean and Japanese studies examined BD associated with MDS, but studies in Chinese patients are rare. Therefore, this study aimed to investigate the characteristics of Chinese patients with BD-MDS and explore the role played by trisomy 8. The results suggest that GI ulceration is common in patients with BD-MDS. Cytogenetic aberration, especially trisomy 8, may play a role in the pathogenesis of intestinal involvement in patients with BD and MDS.

The prevalence of concurrent BD-MDS ranges from 0.4% to 3.1% [26, 33] and has been reported mainly in Japan and Korea [18, 20, 40–44]. In the present study, the prevalence of BD-associated MDS was 2%, with these patients having a high frequency of GI involvement compared to the general BD population. This finding is supported by the data from the literature review. Patients with GI BD and MDS had a higher frequency of ulcers in the ileocecal region than those without MDS, but otherwise the features were similar between the two groups. Thus, MDS appears to increase the risk of GI involvement in patients with BD and also to modify the clinical expression of GI involvement.

According to previous studies, the prevalence of trisomy 8 is only 7–9% in MDS patients with chromosomal abnormalities [21, 22]. The prevalence of trisomy 8 in our patients with BD and MDS was 81.3%, markedly higher than that in patients with MDS alone. Furthermore, the prevalence of GI involvement observed for patients with BD and MDS was consistent with previous literature (62.5% versus 77.3%), higher than that in patients with BD but without MDS (62.5% versus 11.9%) in the present study.

Similar results were obtained when GI BD with or without MDS and with or without trisomy 8 were compared. Patients with GI BD, MDS, and trisomy 8 were more likely to be female, to be older, and to have fever. They were also likely to have lower leukocyte count, hemoglobin level, and platelet count and higher ESR than those without trisomy 8. Therefore, these results strongly suggest that both trisomy 8 and MDS may be responsible for the high frequency of GI involvement in these patients. It is also possible that the two act synergistically. Previous reports have indicated that BD-MDS may constitute a subset distinct from common BD or MDS [18, 20, 25, 30].

Although a relationship was seen between trisomy 8 and symptoms related to BD in the present study and in the literature, the underlying mechanisms are unclear. MDS and BD may have similar pathophysiology. Chen et al. [45] analyzed various gene expression patterns in hematopoietic progenitor cells obtained from patients with MDS and trisomy 8 using microarray analysis. Interestingly, they observed distinctively higher gene expression of several cytokines, including transforming growth factor (TGF)-*β*, interferon (IFN)-*β*2, interleukin (IL)-6, and IL-7R, which are all involved in immune activity and inflammation [45]. BD is a chronic inflammatory disease with abnormalities in several inflammatory cytokines, including IL-1*β*, IL-6, IL-8, IL-17, IL-18, tumor necrosis factor (TNF)-*α*, and IFN-*γ* [46]. Inflammatory cytokines have also recently been reported to be involved in the pathogenesis of MDS [47]. Ohno et al. [48] investigated a BD-associated MDS patient with trisomy 8 and demonstrated increased production of reactive oxygen species (ROS) in the active phase of BD. Another case study from Japan found higher serum levels of soluble IL-2 receptor (IL-2R), IFN-*γ*, IL-1*β*, IL-6, IL-8, and granulocyte-macrophage colony stimulating factor in a BD-associated MDS patient with trisomy 8 during the active phase [28]. Two other groups [31, 32] found that the IL-7/IL-7R-dependent signaling pathway is involved in both the immune response in the intestinal mucosa and in the development of colitis. As BD-MDS patients have high frequencies of intestinal lesions and trisomy 8 and higher expression of several cytokines controlled by genes located on chromosome 8, it is suggested that trisomy 8 may predispose patients with MDS to BD. Nevertheless, the pathogenic association between BD and MDS is still unclear.

There is no unified treatment approach for BD-MDS. Since inflammation plays a pivotal role in BD-MDS [45–48], the treatment regimens are to increase dosage or to add biological agents such as infliximab in addition to the routine/basic treatment of BD, cyclosporin, and immunosuppressants, which are widely used in BD-MDS [4, 15, 25, 30, 39, 44]. Unfortunately, because of the wide variety of treatments, which are often tentatively tailored to each specific patient, we could not perform any analysis on the matter in the present study. In addition, the literature about the treatment of BD-MDS is mostly case reports [27, 30]. Nevertheless, among our patients, three were improved by treatments, nine were controlled, and four were not improved at all. A prospective trial would be needed to determine the best course of treatment, but the rarity of the disease would probably prevent such a trial from occurring.

One patient from the BD-MDS group (Supplementary Table 1, patient #4) deserves special mention. She was initially diagnosed with intestinal BD. At that time, her blood indices were normal but she was diagnosed with trisomy 8. One year into follow-up, she developed anemia (non-small cell pigmented anemia) and was diagnosed as having MDS-U after morphologic, immunophenotypic, cytogenetic, FISH, and molecular analyses. There are reports that immunosuppressive drugs such as methotrexate, azathioprine, or cyclophosphamide may induce therapy-related MDS [49, 50], but this patient was not treated with any of these drugs before the onset of MDS. Other factors were probably involved. It might be worth investigating whether cytogenetic abnormalities or genetic polymorphisms are present in different clinical subsets of BD in the absence of MDS.

Clinically, the present study suggests that patients with BD and hematological abnormality should be tested for trisomy 8. Indeed, since patients with trisomy 8 are prone to MDS and GI involvement, this could change the clinical management of these patients. Conversely, if a patient with MDS develops recurrent oral ulcers, a diagnosis of BD, particularly GI BD, should be considered and investigated.

This retrospective study has several limitations. A major limitation was that there were only three patients with BD-MDS without trisomy 8 and six without GI involvement. With such small numbers it was not possible to establish the importance of trisomy 8 in BD-MDS. Trisomy 8 is not an aneuploidy that is routinely screened for without clinical indication of doing so. Therefore, we could not investigate the patients with trisomy 8 without BD. In addition, screening for trisomy 8 was done on a case-by-case basis and according to the physicians' experience. The exact reasons for screening were not accurately recorded in the medical charts. A large prospective study is needed to confirm our findings. In addition, we did not search for MDS patients with trisomy 8 for inflammatory bowel disease with or without BD.

## 5. Conclusions

In conclusion, patients with BD and MDS are characterized by older age, fever, more frequent GI involvement, lower leukocyte count, and higher ESR level. Cytogenetic abnormalities, especially trisomy 8, might have an important role in BD-associated MDS and intestinal lesions. BD associated with MDS may be a new clinical disease entity, distinct from BD and autoimmune manifestations of MDS. Additional studies are required to ascertain the pathological link between BD-MDS and cytogenetic abnormalities, especially trisomy 8. For now, the results do not support a systematic screening of trisomy 8 in BD with intestinal involvement, but the possibility should be kept in mind.

## Figures and Tables

**Table 1:** Clinical characteristics of patients with BD and/without MDS.

	BD with MDS (*n* = 16)	BD without MDS (*n* = 789)	*P*
Age at BD diagnosis (y), mean (SD)	49.9 ± 12.4	37.9 ± 12.8	0.007^*∗*^
Age at MDS diagnosis (y), mean (SD)	47.5 ± 12.2	-	-
Male (%)	4 (25.0)	409 (51.8)	0.040^*∗*^
Oral ulcer	16 (100)	777 (98.5)	0.821
Genital ulcer	14 (87.5)	558 (70.7)	0.432
Ocular lesion	0 (0)	102 (12.9)	0.391
Arthritis	5 (31.3)	178 (22.6)	0.714
Skin lesions	7 (43.8)	510 (64.6)	0.084
Positive pathergy test	3 (18.8)	55 /125 (44.0)	0.062
Central nervous system	0 (0)	14 (1.8)	>0.99
Vascular lesions	0 (0)	31 (3.9)	>0.99
GI involvement	10 (62.5)	94 (11.9)	<0.001^*∗*^
Fever	9 (56.3)	7 (1.9)	<0.001^*∗*^
Laboratory findings at diagnosis of BD			
CRP (mg/L)	54.8 ± 40.8	12.74 ± 22.16	0.002^*∗*^
ESR (mm/h)	80.2 ± 29.7	23.74 ± 24.38	<0.001^*∗*^
WBC (10^9^/L)	3.6 ± 2.2	7.2 ± 2.5	<0.001^*∗*^
Hemoglobin (g/L)	88.8 ± 18.7	135.6 ± 19.4	<0.001^*∗*^
Platelets (10^9^/L)	144.0 ± 109.0	228.5 ± 75.9	0.016^*∗*^

^*∗*^ *P* < 0.05. BD: Behçet disease; GI: gastrointestinal; CRP: C-reactive protein; ESR: erythrocyte sedimentation rate; WBC: white blood cells.

**Table 2:** Symptoms and laboratory findings in patients with intestinal BD according to MDS.

	Intestinal BD with MDS (*n* = 10)	Intestinal BD without MDS (*n* = 94)	*P*
Age at diagnosis of intestinal BD (years)	49.5 ± 12.8	37.7 ± 13.7	0.008^*∗*^
Male (%)	2 (20.0)	54 (57.4)	0.041^*∗*^
Duration (months)	178 ± 58	107 ± 126	0.129
Systemic signs			
Oral ulcer	10 (100)	90 (95.7)	>0.99
Genital ulcer	8 (80.0)	66 (70.2)	0.708
Ocular lesion	0 (0)	3 (3.2)	>0.99
Arthritis	3 (30.0)	22 (23.4)	0.694
Skin lesions	4 (40.0)	42 (44.7)	>0.99
Positive pathergy test	3 (30.0)	20 (21.3)	0.608
Central nervous system	0 (0)	0 (0)	-
Symptoms of GI involvement			
Abdominal pain	7 (70.0)	82 (87.2)	0.184
Melena/hematochezia	4 (40.0)	30 (31.9)	0.717
Diarrhea	5 (50.0)	46 (48.9)	>0.99
Weight loss	8 (80.0)	44 (46.8)	0.083
Fever	6 (60.0)	8 (8.5)	0.002^*∗*^
Perforation	0 (0)	8 (8.5)	>0.99
Ileus	1 (10.0)	8 (8.5)	>0.99
Location of intestinal ulcers			
Esophagus	0 (0)	18 (19.1)	0.336
Ileocecal	9 (90.0)	46 (48.9)	0.032^*∗*^
Small intestine	1 (10.0)	6 (6.4)	0.548
Colon	1 (10.0)	24 (25.5)	0.426
Laboratory findings at BD diagnosis			
CRP (mg/L)	63.3 ± 41.4	20.7 ± 28.4	0.010^*∗*^
ESR (mm/h)	91.9 ± 21.5	129.4 ± 21.9	<0.001^*∗*^
WBC (10^9^/L)	3.7 ± 2.5	6.8 ± 2.9	0.003^*∗*^
Hemoglobin (g/L)	89.7 ± 17.7	129.4 ± 19.9	<0.001^*∗*^
Platelets (10^9^/L)	163.9 ± 116.4	243.1 ± 92.7	0.023^*∗*^

^*∗*^ *P* < 0.05. BD: Behçet disease; GI: gastrointestinal; CRP: C-reactive protein; ESR: erythrocyte sedimentation rate; WBC: white blood cells.

**Table 3:** Symptoms and laboratory findings in patients with intestinal BD according to trisomy 8.

	Intestinal BD with trisomy 8 (*n* = 10)	Intestinal BD without trisomy 8 (*n* = 10)	*P*
Age at diagnosis of intestinal BD (years)	49.5 ± 12.8	30.7 ± 12.9	0.003^*∗*^
Male (%)	2 (20.0)	5 (50.0)	0.348
Duration (months)	158 ± 50	40 ± 13	0.058
Systemic signs			
Oral ulcer	10 (100)	10 (100)	-
Genital ulcer	8 (80.0)	7 (70.0)	>0.99
Ocular lesion	0 (0)	0 (0)	-
Arthralgia	3 (30.0)	3 (30.0)	>0.99
Skin lesions	4 (40.0)	3 (30.0)	>0.99
Positive pathergy test	3 (30.0)	2 (20.0)	>0.99
Central nerve system	0 (0)	0 (0)	-
Symptoms of GI involvement			
Abdominal pain	7 (70.0)	8 (80.0)	>0.99
Melena/hematochezia	4 (40.0)	5 (50.0)	>0.99
Diarrhea	5 (50.0)	6 (60.0)	>0.99
Weight loss	8 (80.0)	5 (50.0)	>0.99
Fever	6 (60.0)	1 (10.0)	>0.99
Perforation	0 (0)	3 (30.0)	>0.99
Ileus	1 (10.0)	3 (30.0)	>0.99
Location of intestinal ulcers			
Esophagus	0 (0)	1 (10.0)	>0.99
Ileocecal	9 (90.0)	6 (60.0)	0.302
Small intestine	1 (10.0)	2 (20.0)	0.323
Colon	1 (10.0)	2 (20.0)	>0.99
Laboratory findings at BD diagnosis			
CRP (mg/L)	63.3 ± 41.4	44.5 ± 51.5	0.381
ESR (mm/h)	91.9 ± 21.5	39.2 ± 27.4	<0.001^*∗*^
WBC (10^9^/L)	3.7 ± 2.5	8.5 ± 4.0	0.006^*∗*^
Hemoglobin (g/L)	89.7 ± 17.7	111.6 ± 15.3	0.008^*∗*^
Platelets (10^9^/L)	163.9 ± 116.4	339.1 ± 136.6	0.006^*∗*^

^*∗*^ *P* < 0.05. BD: Behçet disease; CRP: C-reactive protein; ESR: erythrocyte sedimentation rate; WBC: white blood cell.

## Supplementary Materials

Supplementary Materials Supplementary Table 1: clinical characteristics of 16 patients with Behçet disease and myelodysplastic syndromes. Supplementary Table 2: comparison between the patients of the present study and those from the literature. Supplementary Table 3: comparison between the patients with trisomy 8 of the present study and those from the literature.

## References

[1] Franks A., Mendelsohn S., Ewins D., Price L., O'Mahoney C. (1998). Behcet's syndrome - Eventually [1]. *International Journal of STD & AIDS*.

[2] Sakane T., Takeno M., Suzuki N., Inaba G. (1999). Behcet's disease. *The New England Journal of Medicine*.

[3] Esatoglu S. N., Hatemi G., Salihoglu A., Hatemi I., Soysal T., Celik A. F. (2015). A reappraisal of the association between Behçet's disease, myelodysplastic syndrome and the presence of trisomy 8: A systematic literature review. *Clinical and Experimental Rheumatology*.

[4] Kaklamani V. G., Vaiopoulos G., Kaklamanis P. G. (1998). Behcet's disease. *Seminars in Arthritis and Rheumatism*.

[5] Zouboulis C. C., Kötter I., Djawari D. (1997). Epidemiological Features of Adamantiades-Behçet's Disease in Germany and in Europe. *Yonsei Medical Journal*.

[6] Shimizu T., Ehrlich G. E., Inaba G., Hayashi K. (1979). Behçet disease (Behçet syndrome). *Seminars in Arthritis and Rheumatism*.

[7] Bayraktar Y., Özaslan E., Van Thiel D. H. (2000). Gastrointestinal manifestations of Behcet's disease. *Journal of Clinical Gastroenterology*.

[8] Lehner T. (1977). Oral ulceration and Behçet's syndrome.. *Gut*.

[9] Cheon J. H., Celik A. F., Kim W. H. (2010). Behcet's disease: gastrointestinal involvement. *Behcet's Syndome*.

[10] Yurdakul S., Tüzüner N., Yurdakul I., Hamuryudan V., Yazici H. (1996). Gastrointestinal involvement in Behçet's syndrome: a controlled study. *Annals of the Rheumatic Diseases*.

[11] Iwata S., Saito K., Yamaoka K. (2011). Efficacy of combination therapy of anti-TNF-*α* antibody infliximab and methotrexate in refractory entero-Behçet's disease. *Modern Rheumatology*.

[12] Garcia-Manero G. (2014). Myelodysplastic syndromes: 2014 update on diagnosis, risk-stratification, and management. *American Journal of Hematology*.

[13] Okamoto T., Okada M., Mori A. (1997). Correlation between immunological abnormalities and prognosis in myelodysplastic syndrome patients. *International Journal of Hematology*.

[14] Marisavljević D., Kraguljac N., Rolović Z. (2006). Immunologic abnormalities in myelodysplastic syndromes: Clinical features and characteristics of the lymphoid population. *Medical Oncology*.

[15] Giannouli S., Voulgarelis M., Zintzaras E., Tzioufas A. G., Moutsopoulos H. M. (2004). Autoimmune phenomena in myelodysplastic syndromes: a 4-yr prospective study. *Rheumatology*.

[16] de Hollanda A., Beucher A., Henrion D. (2011). Systemic and immune manifestations in myelodysplasia: a multicenter retrospective study. *Arthritis Care & Research*.

[17] Kimura S., Kuroda J., Akaogi T., Hayashi H., Kobayashi Y., Kondo M. (2001). Trisomy 8 Involved in Myelodysplastic Syndromes as a Risk Factor for Intestinal Ulcers and Thrombosis — Behçet's Syndrome. *Leukemia & Lymphoma*.

[18] Tada Y., Koarada S., Haruta Y., Mitamura M., Ohta A., Nagasawa K. (2006). The association of Behçet's disease with myelodysplastic syndrome in Japan: a review of the literature. *Clinical and Experimental Rheumatology*.

[19] Papageorgiou A., Ziakas P. D., Tzioufas A. G., Voulgarelis M. (2013). Indications for bone marrow examination in autoimmune disorders with concurrent haematologic alterations. *Clinical and Experimental Rheumatology*.

[20] Kawabata H., Sawaki T., Kawanami T. (2006). Myelodysplastic syndrome complicated with inflammatory intestinal ulcers: Significance of trisomy 8. *Internal Medicine*.

[21] Sole F., Espinet B., Sanz G. F., et al. (2000). Incidence, characterization and prognostic significance of chromosomal abnormalities in 640 patients with primary myelodysplastic syndromes. Grupo Cooperativo Espanol de Citogenetica Hematologica. *British Journal of Haematology*.

[22] Bernasconi P., Klersy C., Boni M. (2005). Incidence and prognostic significance of karyotype abnormalities in de novo primary myelodysplastic syndromes: A study on 331 patients from a single institution. *Leukemia*.

[23] Mora P., Avellis F. O., Zavota L., Orsoni J. G. (2008). Behçet's disease associated with trisomy 8 in a young Italian girl - A case report. *Clinical and Experimental Rheumatology*.

[24] Becker K., Fitzgerald O., Green A. J. (2009). Constitutional trisomy 8 and behcet syndrome. *American Journal of Medical Genetics Part A*.

[25] Ahn J. K., Cha H.-S., Koh E.-M. (2008). Behcet's disease associated with bone marrow failure in Korean patients: clinical characteristics and the association of intestinal ulceration and trisomy 8. *Rheumatology*.

[26] Ahn J. K., Oh J. M., Lee J., Koh E.-M., Cha H.-S. (2010). Behcet's disease associated with malignancy in Korea: A single center experience. *Rheumatology International*.

[27] Kimura M., Tsuji Y., Iwai M. (2015). Usefulness of adalimumab for treating a case of intestinal Behçet's disease with trisomy 8 myelodysplastic syndrome. *Intestinal Research*.

[28] Hasegawa H., Iwamasa K., Hatta N., Fujita S. (2003). Behçet's disease associated with myelodysplastic syndrome with elevated levels of inflammatory cytokines. *Modern Rheumatology*.

[29] Kawano S., Hiraoka S., Okada H., Akita M., Iwamuro M., Yamamoto K. (2015). Clinical features of intestinal Behçet's disease associated with myelodysplastic syndrome and trisomy 8. *Acta Medica Okayama*.

[30] Toyonaga T., Nakase H., Matsuura M. (2013). Refractoriness of intestinal Behçet's disease with Myelodysplastic syndrome involving Trisomy 8 to medical therapies -our case experience and review of the literature. *Digestion*.

[31] Watanabe M., Ueno Y., Yajima T. (1998). Interleukin 7 transgenic mice develop chronic colitis with decreased interleukin 7 protein accumulation in the colonic mucosa. *The Journal of Experimental Medicine*.

[32] Yamazaki M., Yajima T., Tanabe M. (2003). Mucosal T cells expressing high levels of IL-7 receptor are potential targets for treatment of chronic colitis. *The Journal of Immunology*.

[33] Lin Y., Li G., Zheng W., Tian X., Zhang F. (2014). Behcet's disease associated with malignancy: A report of 41 Chinese cases. *International Journal of Rheumatic Diseases*.

[34] International Study Group for Behçet's Disease (1990). Criteria for diagnosis of Behçet's disease. *The Lancet*.

[35] The International Criteria for Behcet's Disease (ICBD) (2014). A collaborative study of 27 countries on the sensitivity and specificity of the new criteria. *Journal of the European Academy of Dermatology and Venereology*.

[36] Vardiman J. (2012). The classification of MDS: from FAB to WHO and beyond. *Leukemia Research*.

[37] Garcia J. R. G., Meza-Espinoza J. P. (2006). Use of the International System for Human Cytogenetic Nomenclature (ISCN) [8]. *Blood*.

[38] Chun K., Hagemeijer A., Iqbal A., Slovak M. L. (2010). Implementation of standardized international karyotype scoring practices is needed to provide uniform and systematic evaluation for patients with myelodysplastic syndrome using IPSS criteria: An International Working Group on MDS Cytogenetics Study. *Leukemia Research*.

[39] Kook M.-H., Yhim H.-Y., Lee N.-R. (2014). Successful treatment of myelodysplastic syndrome and behcet colitis after allogeneic hematopoietic stem cell transplantation. *Korean Journal of Internal Medicine*.

[40] Fujimura T., Yukawa N., Nakashima R. (2010). Periodic fever and erythema nodosum associated with MDS with trisomy 8: report of two cases and review of the literature.. *Modern rheumatology / the Japan Rheumatism Association*.

[41] Lin Y.-C., Liang T.-H., Chang H.-N., Lin J.-S., Lin H.-Y. (2008). Behçet disease associated with myelodysplastic syndrome. *JCR: Journal of Clinical Rheumatology*.

[42] Mantzourani M. G., Chantziara K., Thanopoulou I., Variami H., Vaiopoulos G., Pangalis G. A. (2009). Coexistence of Behcet's disease and chronic myelomonocyte leukemia with trisomy 8: a case report and review of the literature. *Clinical and Experimental Rheumatology*.

[43] Kovacs E., Nemeth H., Telek B. (2009). Behçet's disease in a patient with myelodysplastic syndrome. *Clinical Lymphoma, Myeloma & Leukemia*.

[44] Cengiz M., Altundag M. K., Zorlu A. F., Güllü I. H., Özyar E., Atahan I. L. (2001). Malignancy in Behçet's disease: A report of 13 cases and a review of the literature. *Clinical Rheumatology*.

[45] Chen G., Zeng W., Miyazato A. (2004). Distinctive gene expression profiles of CD34 cells from patients with myelodysplastic syndrome characterized by specific chromosomal abnormalities. *Blood*.

[46] Hamzaoui K., Hamzaoui A., Guemira F., Bessioud M., Hamza M., Ayed K. (2002). Cytokine profile in Behçet's disease patients: relationship with disease activity. *Scandinavian Journal of Rheumatology*.

[47] Sloand E. M., Barrett A. J. (2010). Immunosuppression for myelodysplastic syndrome: How bench to bedside to bench research led to success. *Hematology/Oncology Clinics of North America*.

[48] Ohno E., Ohtsuka E., Watanabe K. (1997). Behcet's disease associated with myelodysplastic syndromes: A case report and a review of the literature. *Cancer*.

[49] Pedersen-Bjergaard J., Andersen M. K., Christiansen D. H., Nerlov C. (2002). Genetic pathways in therapy-related myelodysplasia and acute myeloid leukemia. *Blood*.

[50] Kwong Y. L., Au W. Y., Liang R. H. S. (1998). Acute myeloid leukemia after azathioprine treatment for autoimmune diseases: Association with -7/7q-. *Cancer Genetics and Cytogenetics*.

