# Supplementary material for: High Incidence of Gastrointestinal Ulceration and Cytogenetic Aberration of Trisomy 8 as Typical Features of Behçet's Disease Associated with Myelodysplastic Syndrome: A Series of 16 Consecutive Chinese Patients from the Shanghai Behçet's Disease Database and Comparison with the Literature
Source: Biomed Res Int. 2018 Apr 24;2018:8535091. doi: 10.1155/2018/8535091 (PMC5941803; doi:10.1155/2018/8535091)
Supplement: Supplementary Materials — Supplementary Table 1: clinical characteristics of 16 patients with Behçet disease and myelodysplastic syndromes. Supplementary Table 2: comparison between the patients of the present study and those from the literature. Supplementary Table 3: comparison between the patients with trisomy 8 of the present study and those from the literature. [file 8535091.f1.doc]

Supplementary Table 1. Clinical characteristics of 16 patients with Behçet disease and myelodysplastic syndromes.

| Case No. | Age/sex | BD symptoms | MDS type | Karyotype and FISH | Endoscopic findings | Distribution pattern | BD duration (years) | MDS duration (years) | Treatment | Cytotoxic Tx prior to MDS | Outcome of treatments |
| --- | --- | --- | --- | --- | --- | --- | --- | --- | --- | --- | --- |
| 1 | 47/F | IOGA | MDS-RCMD | 47XX+8 | Terminal ileum, rectum, CU | Multi-segmental | 20 | 20 | Prednisone  Cyclosporine  Thalidomide  Infliximab | - | Improved |
| 2 | 36/F | IOGS | MDS-U | 47XX+8 | Terminal ileum | Multi-segmental | 6 | 10 | Prednisone  Cyclosporine  Thalidomide  Infliximab | - | Improved |
| 3 | 35/M | IOG | MDS-U | 47XX+8 | Terminal ileum | Single | 5 | 1 | Prednisone  Infliximab | Cyclosporine Thalidomide | Not improved |
| 4 | 71/F | IOSA | MDS-U | 47XX+8 | Terminal ileum, ileocecal valve | Multi-segmental | 40 | 1 | Prednisone  Cyclosporine  Infliximab | MMF Thalidomide | Not improved |
| 5 | 46/F | IOGPA | MDS-U | 47XX+8 | Ileocecal valve | Single | 10 | 10 | Prednisone  Cyclosporine  Thalidomide  Infliximab | - | Stable |
| 6 | 66/M | IOGS | MDS-RAEB | 48,XY,+8,+19,del(20)(q11.2) | Terminal ileum, CU | Multi-segmental | 5 | 0.5 | Prednisone  Cyclosporine  Thalidomide  Infliximab | - | Not improved |
| 7 | 58/F | IOPS | MDS-RCUD-RN | 47XX+8 | Ileocecal valve | Single | 14 | 20 | Prednisone  Cyclosporine  Thalidomide  Infliximab | - | Improved |
| 8 | 49/F | IOG | MDS-RCMD | 47XX+8 | Terminal ileum, CU，small intestine | Multi-segmental | 36 | 20 | Prednisone  Cyclosporine  Infliximab | Thalidomide | Stable |
| 9 | 34/F | IOGP | MDS-RCMD | 47XX+8 | Ileocecal valve | Single | 8 | 7 | Prednisone  Cyclosporine  Thalidomide  Infliximab | - | Stable |
| 10 | 58/F | IOG | MDS-RCUD | 47XX+8,-20,add(21)(q21) | Terminal ileum, CU | Multi-segmental | 4 | 4 | Prednisone  Cyclosporine  Thalidomide  Infliximab | - | Not improved |
| 11 | 36/F | OGSA | MDS-U | 47XX+8 | - | - | 3 | 3 | Prednisone  Cyclosporine  Thalidomide | - | Stable |
| 12 | 48/F | OGS | MDS-U | 47XX+8 | - | - | 10 | 3 | Prednisone  Cyclosporine  Thalidomide | Thalidomide | Stable |
| 13 | 58/M | OGS | MDS-RAEB | 47XX+8 | - | - | 3 | 3 |  | - | Stable |
| 14 | 36/M | OGS | MDS-RAEB | Normal | - | - | 5 | 5 | Prednisone  Cyclosporine  Thalidomide | - | Stable |
| 15 | 58/F | OGA | MDS- RCMD | Normal | - | - | 6 | 10 | Prednisone  Cyclosporine  Thalidomide | - | Stable |
| 16 | 60/F | OGS | MDS-U | Normal | - | - | 7 | 2 | Prednisone  Cyclosporine  Thalidomide | Thalidomide | Stable |

I: Intestinal involvement; O: oral ulcer; G: genital ulcer; S: skin lesion; A: arthritis; P: pathergy. MDS-U: MDS-unclassifiable, RCMD: refractory cytopenia with multilineage dysplasia, RAEB: refractory anemia with excess of blasts, RCUD: refractory cytopenia with unilineage dysplasia. CU: colonic ulcers. MMF: mycophenolate mofetil.

Supplementary Table 2. Comparison between the patients of the present study and those from the literature.

|  | BD-MDS (n = 16) (our group) | BD-MDS (n = 57)  (in literature) | P |
| --- | --- | --- | --- |
| Age at BD diagnosis (years), mean (SD) | 49.9 ± 12.4 | 48.7 ± 18.0 | 0.822 |
| Age at MDS diagnosis (years), mean(SD) | 47.5 ± 12.2 | 47.4 ± 16.9 | 0.994 |
| Male (%) | 4 (25.0) | 30 (52.6) | 0.069 |
| Oral ulcer | 16 (100) | 56 (98.2) | >0.99 |
| Genital ulcer | 14 (87.5) | 44 (77.2) | 0.720 |
| Ocular lesion | 0 (0) | 3 (5.3) | >0.99 |
| Arthritis | 5 (31.3) | 17 (29.8) | >0.99 |
| Skin lesions | 7 (43.8) | 37 (64.9) | 0.409 |
| Positive pathergy test | 3 (18.8) | 14/25(56.0) | 0.025 |
| Central nervous system | 0 (0) | 2 (3.50) | >0.99 |
| Vascular lesions | 0 (0) | 6 (10.5) | 0.585 |
| GI involvement | 10 (62.5) | 41 (71.9) | 0.468 |
| Fever | 9 (56.3) | 36 (63.2) | 0.545 |
| Trisomy 8 | 13 (81.3) | 44 (77.2) | 0.729 |

**Supplementary Table 3. Comparison between the patients with trisomy 8 of the present study and those from the literature.**

|  | BD-MDS with trisomy 8 (n = 13)  (our group) | BD-MDS with trisomy 8 (n = 44)  (in literature) | P |
| --- | --- | --- | --- |
| Age at BD diagnosis (years), mean (SD) | 49.5 ± 12.8 | 49.5 ± 18.3 | 0.997 |
| Age at MDS diagnosis (years ), mean (SD) | 48.7 ± 13.3 | 48.2 ± 17.4 | 0.933 |
| Male (%) | 3 (20.0) | 22 (50.0) | 0.157 |
| Oral ulcer | 13 (100) | 44 (100) | - |
| Genital ulcer | 11 (84.6) | 35 (79.5) | >0.99 |
| Ocular lesion | 0 (0) | 3 (6.8) | >0.99 |
| Arthritis | 4 (30.8) | 10 (22.7) | 0.715 |
| Skin lesions | 5 (38.5) | 25 (56.8) | 0.244 |
| Positive pathergy test | 3 (23.1) | 12/22 (54.5) | 0.089 |
| Central nervous system | 0 (0) | 0 (0) | - |
| Vascular lesions | 0 (0) | 6 (13.6) | 0.580 |
| GI involvement | 10 (76.9) | 34 (77.3) | 0.178 |
| Fever | 6 (60.0) | 32 (72.7) | 0.459 |
